# Generation of human induced pluripotent stem cell lines from patients with *FGFR2*-linked syndromic craniosynostosis

**DOI:** 10.1242/dmm.052123

**Published:** 2025-09-18

**Authors:** Max Gijsbertsen, Irene M. J. Mathijssen, Ana F. Duarte Madancos, Johannes P. T. M. van Leeuwen, Jeroen van de Peppel

**Affiliations:** ^1^ Erasmus MC, University Medical Center Rotterdam, Department of Internal Medicine, Dr. Molewaterplein 40, 3015 GD Rotterdam, The Netherlands; ^2^ Erasmus MC, University Medical Center Rotterdam, Department of Plastic and Reconstructive Surgery, Dr. Molewaterplein 40, 3015 GD Rotterdam, The Netherlands

**Keywords:** Craniosynostosis, FGFR2, Crouzon, Apert, Pfeiffer, hiPSC

## Abstract

Craniosynostosis is a multigenic congenital condition in which one or more calvarial sutures have prematurely fused during the development of the fetus. Pathogenic variants in *FGFR2* are associated with the development of syndromic craniosynostosis, such as Crouzon, Apert and Pfeifer syndromes. Investigation of *FGFR2*-linked craniosynostosis is hindered by the lack of appropriate *in vitro* models. Patient-derived human induced pluripotent stem cell (hiPSC) *in vitro* disease models provide the opportunity to investigate the disease, identify molecular targets for pharmaceutical treatments, and enable the generation of autologous pluripotent stem cell catalogues. Here, we report three patient-derived hiPSC lines carrying the C342Y, S252W or E565G FGFR2 pathogenic variant. The patient hiPSC lines express characteristic pluripotency markers and display distinct phosphorylation profiles under unstimulated conditions. FGFR2^C342Y^ showed autophosphorylation in the absence of bFGF ligand, although downstream docking proteins PLCγ and FRS2α were not phosphorylated. FGFR2^S252W^ and FGFR2^E565G^ hiPSCs showed increased phosphorylation of docking proteins PLCγ and FRS2α, whereas FGFR2 was not phosphorylated. These patient hiPSC lines provide molecular and cellular options to investigate *FGFR2*-linked craniosynostosis in the patient-specific genomic context and develop therapeutic modalities.

## Teaser

**Summary:** This article emphasizes the utility of patient-derived stem cell *in vitro* disease models in the investigation of *FGFR2*-linked craniosynostosis and describes distinct differences in FGFR2 signaling within this condition.

## INTRODUCTION

The mesenchymal sutures that separate the bony plates of the calvarial vault enable flexibility of the skull during birth and postnatal development. The calvarial plates gradually ossify outwards from their osteogenic fronts but do not completely fuse together until adult age (Cohen, 1993; Jin et al., 2016). Craniosynostosis is a congenital condition in which the mesenchymal sutures between the calvarial plates fuse prematurely during the development of the fetus and within the first year after birth. Consequently, the skull is unable to expand in tandem with the developing brain (Ciurea and Toader, 2009; Morriss-Kay and Wilkie, 2005; Posnick and Ruiz, 2000). Premature fusion may occur within single or multiple sutures, depending on the syndrome and associated pathogenic variants (Kimonis et al., 2007; Twigg and Wilkie, 2015; Wenger et al., 1993; Wilkie, 1997; Wilkie et al., 2017). As a result, the patients present with aberrant skull growth and are at risk for raised intracranial pressure (Mathijssen, 2021; Posnick and Ruiz, 2000). Patients often require multiple surgical interventions, of which the first intervention is commonly performed within the first year after birth (Bergquist et al., 2016). To date, surgical interventions are the sole treatment to successfully and sufficiently relieve patients from the increasing cranial pressure and allow the brain to naturally progress its development (Mathijssen, 2021). Additionally, facial anomalies commonly described in craniosynostosis patients are adjusted during surgery as well (Levi et al., 2012; Marchac and Renier, 1989).

Craniosynostosis is a consequence of genetic variants as well as exposure to environmental risk factors and has been associated with over 180 syndromes as well as non-syndromic cases (Durham et al., 2017). On a genetic level, craniosynostosis is associated with a variety of pathogenic variants in over 60 genes (Goos and Mathijssen, 2019; Tooze et al., 2023; Wilkie et al., 2017). Pathogenic variants in one of the most frequently affected genes, fibroblast growth factor receptor 2 (*FGFR2*), are associated with a multitude of phenotypically distinct syndromes, such as Crouzon, Apert and Pfeiffer syndromes, among others (Anderson et al., 1998; Kan et al., 2002; Kimonis et al., 2007; Neilson and Friesel, 1995; Wenger et al., 1993). FGFR2 signaling is critically involved in many cellular processes during development, including cell proliferation, osteogenic differentiation and cell survival (Itoh and Ornitz, 2004; Kimonis et al., 2007; Wilkie et al., 1995). FGFR2 can stimulate signaling to various downstream pathways, including PLCγ/PKC, PI3K/AKT and MEK/MAPK pathways (Ornitz and Itoh, 2015). The signaling of these pathways is initiated through the binding and subsequent phosphorylation of docking proteins by FGFR2 tyrosine kinase activity. Phosphorylation of docking proteins phospholipase C-gamma (PLCγ) and fibroblast growth factor receptor substrate 2 alpha (FRS2α) results in the canonical activation of the PKC pathway, or the PI3K and MAPK pathway, respectively. Pathogenic variants in FGFR2 can impact and alter the way in which downstream signaling is mediated (Miraoui et al., 2009). Although many publications provide well-defined functional effects of various pathogenic variants on FGFR2 function, these studies are often performed on non-human models or with overexpression constructs of mutant *FGFR2* alleles (Ahmed et al., 2008; Anderson et al., 1998; Eswarakumar et al., 2006; Liu et al., 2013; Mangasarian et al., 1997; Mansukhani et al., 2000; Miraoui et al., 2009; Neilson and Friesel, 1995; Snyder-Warwick et al., 2010; Suzuki et al., 2012; Yu et al., 2000). Currently, investigation of the etiology of *FGFR2*-linked craniosynostosis is hindered by the lack of appropriate *in vitro* disease models that can properly mimic the disease.

Patient-derived human induced pluripotent cell (hiPSC) models are a valuable tool to investigate the consequences of a variant in an *in vitro* model as they can be differentiated into cells that may reflect the affected tissue of the patient itself. The use of patient-derived hiPSCs brings many advantages (Al Abbar et al., 2020; Rispoli et al., 2024; Walia et al., 2012). First, the utilized cell lines retain the cellular context and genetic makeup of the patient, thereby reflecting the patient much more closely than any transgenic or genome-edited line would do. Second, because these lines harbor the pathogenic variant of interest, they do not require any transgenic (over)expression approaches to establish an *in vitro* disease model. Third, reprogramming of the cells can be done with various tissues [e.g. fibroblasts and peripheral blood mononuclear cells (PBMCs)], creating many minimally invasive ways of obtaining source material. Fourth, the cell lines are autologous and circumvent immunological responses involved in transplantation approaches. Last, hiPSCs provide a unique opportunity to obtain a catalogue of pluripotent cells from full adult patients that generally lack pluripotent stem cell niches. Pluripotent cells can be precisely differentiated towards many different lineages, creating tissue types that would normally be hard to obtain for research or transplantation. With a catalogue of pluripotent cells, hiPSCs pave the way for many treatment applications that include gene replacement therapy, smaller-scale personalized drug screening, and CRISPR/Cas9 genome editing to correct causative genomic pathogenic variants (Al Abbar et al., 2020; Rispoli et al., 2024).

Here, we report the generation of three hiPSC lines derived from PBMCs of three patients, each harboring a different pathogenic variant in *FGFR2* resulting in Crouzon, Apert or Crouzon/Pfeiffer syndrome. Patient lines will hereafter be referred to as follows: FGFR2^C342Y^ [EMC170i: Crouzon; p.C342Y (c.1025 G>A)], FGFR2^S252W^ [EMC242i: Apert; p.S252W (c.755 C>G)] and FGFR2^E565G^ [EMC247i: Crouzon/Pfeiffer; p.E565G (c.1694 A>G)] (Tables 1 and 2). The hiPSC lines described here can support fundamental research on variant- and patient-specific disease mechanisms, aberrant downstream signaling and alterations in osteogenic differentiation in *FGFR2*-related craniosynostosis. Furthermore, the hiPSCs may serve as a tool for personalized validation of potential curative drugs. Finally, patient-specific hiPSC lines provide us with the opportunity to target causative pathogenic variants for genomic repair and the generation of autologous, pluripotent stem cells with healthy *FGFR2* copies.

**Table 1.** Patient resource details of reported human induced pluripotent stem cell lines

Unique stem cell line identifier	EMC170i; EMC242i; EMC247i
Alternative name(s) of stem cell line	FGFR2^C342Y^; FGFR2^S252W^; FGFR2^E565G^
Institution	iPS Core Facility, Erasmus University Medical Center, Rotterdam, The Netherlands
Contact information of distributor	J.v.d.P. (h.vandepeppel@erasmusmc.nl); M.G. (m.gijsbertsen@erasmusmc.nl)
Type of cell line	Induced pluripotent stem cell
Origin	Human
Additional origin information	
- Age	EMC170i: 2 years
	EMC242i: 11 years
	EMC247i: 10 years
- Sex	EMC170i: Male
	EMC242i: Male
	EMC247i: Male
- Ethnicity	EMC170i: Undisclosed
	EMC242i: Undisclosed
	EMC247i: Undisclosed
Cell source	Peripheral blood mononuclear cells
Clonality	Clonal
Method of reprogramming	CytoTune-iPS 2.0 Sendai Reprogramming. hKlf4, hOct3/4, hSox2; hc-Myc; hKlf4
Genetic modification	Yes
Type of modification	Hereditary
Associated disease	Syndromic (Crouzon; Apert; Crouzon/Pfeiffer) craniosynostosis
Gene/locus	EMC170i: NM_000141.5; FGFR2 [c.1025G>A(p.Cys342Tyr)], germline variant, somatic. GRCh38; 10q26.13
	EMC242i: NM_000141.5; FGFR2 [c.755C>G(p.Ser252Trp)], germline variant, somatic. GRCh38; 10q26.13
	EMC247i: NM_000141.5; FGFR2 [c.1694A>G(p.Glu565Gly)], germline variant, somatic. GRCh38; 10q26.13
Method of modification	N/A
Name of transgene or resistance	N/A
Inducible/constitutive system	N/A
Date archived/stock date	EMC170i: 06-12-2019
	EMC242i: 16-03-2021
	EMC247i: 16-04-2021
Cell line repository/bank	iPS Core Facility Erasmus University Medical Center
Ethical approval	Signed informed consent of patient's guardian(s)

N/A, not applicable.

**Table 2.** Patient cell line overview o**f** respective pathogenic variants, associated syndrome, other patient details and impact on FGFR2 protein activity

hiPSC line	Associated syndrome	Sex	Gene	Genomic variant	Protein	Functional consequence	Patient phenotype
EMC170i	Crouzon	Male	*FGFR2*	c.1025 G>A	p.C342Y	Constitutively phosphorylated receptor (Neilson and Friesel, 1995)	Craniosynostosis of both lamboid sutures, mild exorbitism, mild midface hypoplasia. Normal hands and feet.
						
EMC242i	Apert	Male	*FGFR2*	c.755 C>G	p.S252W	Increased ligand sensitivity and aspecificity (Anderson et al., 1998)	Craniosynostosis of both coronal sutures, mild exorbitism, midface hypoplasia, complex syndactyly of digits 2-3-4-5 on both hands.
						
EMC247i	Crouzon/Pfeiffer	Male	*FGFR2*	c.1694 A>G	p.E565G	Impaired autoinhibition of receptor phosphorylation (Chen et al., 2007)	Postnatal development of pansynostosis, nystagmus, delayed speech and language development, very mild midface hypoplasia. Normal hands and feet.
						

## RESULTS

### Patient hiPSC lines display pluripotency and genomic stability

**Fig. 1.**
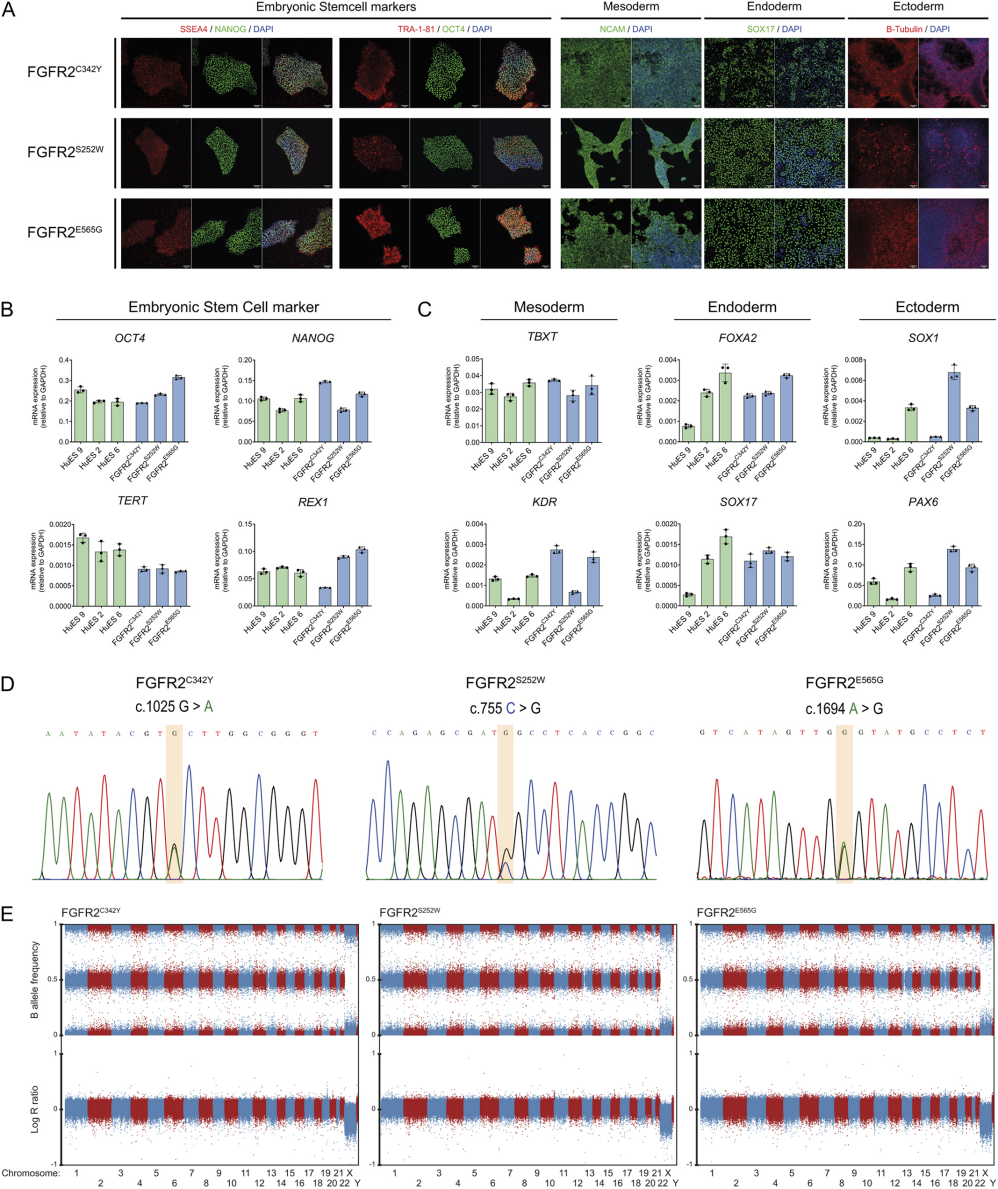
**Identification of pluripotency in patient-derived human induced pluripotent stem cell (hiPSC) lines FGFR2^C342Y^, FGFR2^S252W^ and FGFR2^E565G^.** (A) Immunocytochemistry for embryonic stem cell markers SSEA4, NANOG, TRA-1-81 and OCT-4 (left panel), and trilineage markers NCAM, SOX17 and β-tubulin (right panel), in patient-derived hiPSCs. (B,C) RT-qPCR analysis of trilineage and embryonic stem cell markers of patient-derived hiPSCs. The mRNA expression of embryonic stem cell markers (B) and trilineage markers (C) of multiple human embryonic stem cell (HuES) clones (green bars) are used as reference values for the determination of expressed RNA markers in the reported hiPSC lines FGFR2^C342Y^, FGFR2^S252W^ and FGFR2^E565G^ (blue bars). Gene of interest values are corrected against *GAPDH* reference gene expression. Error bars display mean±s.d. Replicates used in each cell line tested *n*=3. (D) Identification of the respective craniosynostosis variant and genomic stability of the patient-derived hiPSCs. The orange bars highlight the nucleotide location of the patient pathogenic variant. (E) Compressed genomic overview of Global Screening Array analysis of the three patient-derived hiPSC lines FGFR2^C342Y^, FGFR2^S252W^ and FGFR2^E565G^. B-allele frequency and Log(R) ratio are listed on the *y*-axis. Chromosome numbers are listed on the *x*-axis and in the graph in alternating blue and red coloration.

All three hiPSC lines express the embryonic stem cell markers SSEA4, NANOG, TRA-1-81 and OCT4. Tri-lineage commitment towards mesoderm, endoderm and ectoderm was confirmed by protein expression of NCAM (also known as NCAM1), SOX17 and β-tubulin, respectively (Fig. 1A). Reverse transcriptase quantitative polymerase chain reaction (RT-qPCR) analyses showed that each hiPSC line expressed the embryonic stem cell markers *OCT4* (also known as *POU5F1*), *NANOG*, *REX1* (also known as *ZFP42*) and *TERT*. Furthermore, the hiPSC lines expressed lineage-specific marker genes when differentiated towards either mesoderm (*TBXT* and *KDR*), endoderm (*SOX17* and *FOXA2*) or ectoderm (*SOX1* and *PAX6*) (Fig. 1B,C). Three curated human embryonic stem cell (HuES) lines (HuES 2, 6 and 9) were included as pluripotency controls and showed similar signals for embryonic stem cell markers as well as lineage-specific markers to the patient hiPSC lines (Fig. 1B,C).

The presence of the heterozygous *FGFR2* pathogenic variants in the patient hiPSC lines was confirmed by Sanger sequencing (Fig. 1D). Genomic stability of each hiPSC line was assessed by Illumina Global Screening Array (GSA) and showed no copy number variations (CNVs) or large genomic aberrations in FGFR2^C342Y^ (Fig. 1E; Fig. S1A-C). However, a 75 kb duplication on chromosome 10 was observed in FGFR2^S252W^, and a 160 kb deletion on chromosome 13 was observed in FGFR2^E565G^ (Fig. S1D,E). These two large variants are also present in the patient PBMC samples from which the hiPSC are generated and are therefore not due to the reprogramming process (Fig. S1F).

### C342Y-mutated FGFR2 exhibits ligand-independent phosphorylation, whereas S252W- and E565G-mutated FGFR2 remains unphosphorylated, in the absence of receptor-activating ligand

Each patient-derived hiPSC line carries a distinct pathogenic variant that affects FGFR2 protein function differently (Table 2). Wild-type (WT) FGFR2 receptor proteins exhibit a minimal level of receptor phosphorylation in the absence of any stimulating basic fibroblast growth factor (bFGF; also known as FGF2) ligand (Ahmed et al., 2010, 2008; Lin et al., 2012). However, C342Y- and E565G-mutated FGFR2 are described to be constitutively, ligand-independently autophosphorylated (Chen et al., 2007; Neilson and Friesel, 1995). In contrast, S252W-mutated FGFR2 stimulation still requires ligand binding (Anderson et al., 1998; Yu et al., 2000). Therefore, we investigated the unstimulated state of FGFR2 phosphorylation in all three patient hiPSC lines by culturing the cells in bFGF-free ‘TeSR E6’ culture medium for 24 h prior to preparation of protein extracts.

**Fig. 2.**
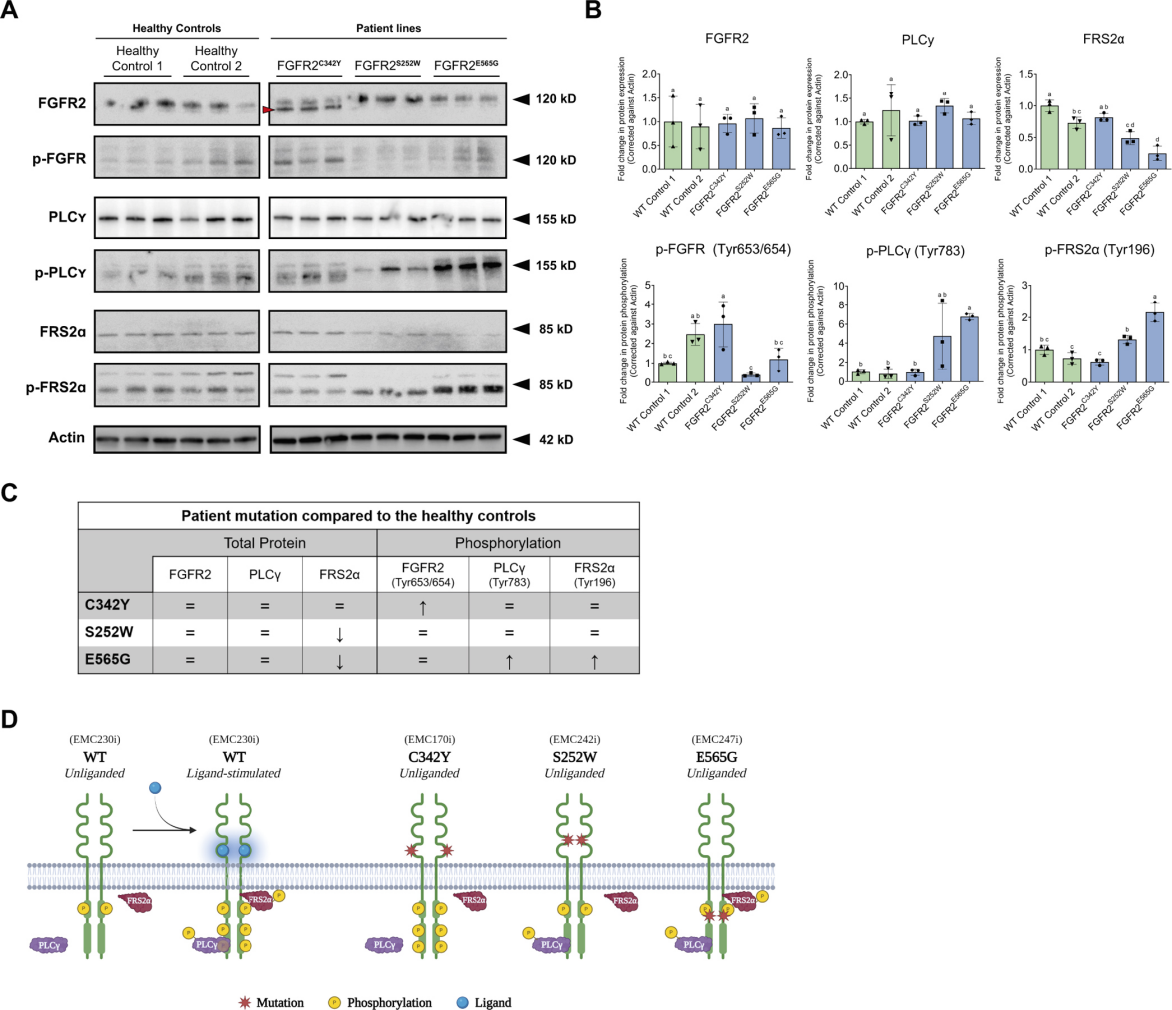
**Protein and phosphorylation analysis of FGFR2 and docking proteins PLCγ and FRS2α in bFGF-free conditions.** (A) Western blot chemiluminescence images of healthy control samples 1 and 2, and patient samples FGFR2^C342Y^, FGFR2^S252W^ and FGFR2^E565G^. For each sample and condition, 10 µg protein-isolated whole-cell lysate is loaded. The red arrowhead indicates the secondary lower-molecular mass band of FGFR2. Technical triplicates (*n*=3) are derived from three parallel, but separate, cultures. (B) Comparative quantification of protein analysis through western blotting of FGFR2, PLCγ, FRS2α and their phosphorylated forms in bFGF-free conditions. Data from healthy control samples 1 and 2 are shown as green bars. Data from patient samples FGFR2^C342Y^, FGFR2^S252W^ and FGFR2^E565G^ are shown as blue bars. Protein values are corrected against actin loading control values. Statistical analysis was performed using one-way ANOVA and Tukey's multiple comparison post-hoc test. Error bars display mean±s.d. Replicates used in each cell line tested *n*=3. Statistical significance is displayed in compact letter display format, with the significance threshold of *P*<0.05. (C) A table summarizing the observations on protein expression and phosphorylation of FGFR2, PLCγ and FRS2α for all three patient lines. Equal marks represent no significant difference compared to healthy controls. Upward- and downward-pointing arrows indicate significant upregulation or downregulation, respectively, compared to healthy control 1. (D) Schematic model speculating how the various patient FGFR2 pathogenic variants impact their phosphorylation and activation of the docking proteins in an unliganded state according to published literature (reviewed in Ornitz and Itoh, 2015) and our findings. WT, wild type. Created in BioRender by Gijsbertsen, M. (2025). https://BioRender.com/dhiibrf. This figure was sublicensed under CC-BY 4.0 terms.

FGFR2 expression was similar in all three patient hiPSC lines and unaltered compared to that in the healthy controls (Fig. 2A-C; Figs S2, S3). A lower-molecular mass fragment was present in the FGFR2^C342Y^ samples, which was absent in both control samples as well as the FGFR2^S252W^ and FGFR2^E565G^ patient samples (Fig. 2A; Figs S2, S3). As expected, we observed increased phosphorylation on unstimulated FGFR tyrosine kinase residues 653/654 in FGFR2^C342Y^ compared to healthy control 1 samples and both FGFR2^S252W^ and FGFR2^E565G^ lines. Both these latter patient hiPSC lines showed an unstimulated phosphorylation state similar to that in the healthy control line 1 (Fig. 2A-C; Fig. S3). These results demonstrate that the FGFR2^C342Y^ hiPSC line shows the characteristic ligand-independent autophosphorylation of the receptor, whereas autophosphorylation of FGFR2 in the other two hiPSC lines is absent.

### Altered phosphorylation of FGFR2 docking proteins PLCγ and FRS2α in the patient hiPSC lines in the absence of bFGF

FGFR2 mediates signal transduction to various downstream pathways, and is initiated through the binding and subsequent phosphorylation of docking proteins PLCγ and FRS2α by FGFR2 tyrosine kinase activity. Our patient pathogenic variants could ultimately lead to unintended effects on the activation of the respective signaling pathways. Therefore, we investigated how the patient pathogenic variants impact the expression and phosphorylation of the FGFR2 docking proteins PLCγ and FRS2α under unstimulated, bFGF-free conditions.

For all three patient hiPSC lines, total protein expression of PLCγ remained unchanged compared to that in the healthy controls. Protein expression of FRS2α did not appear to be directly significantly changed in either of the three patient hiPSC lines, compared to the control (Fig. 2A-C; Fig. S3). Although the data in Fig. 2A-C indicate statistically significant reduced expression between the control samples and FGFR2^S252W^ and FGFR2^E565G^, this was not supported by the supplementary experiment (Fig. S3). Despite the constitutive phosphorylation of FGFR in FGFR2^C342Y^, phosphorylation of both PLCγ and FRS2α was similar to that in the healthy control lines (Fig. 2A-C; Fig. S3). In FGFR2^S252W^, we observed a trend in which PLCγ and FRS2α phosphorylation appeared substantially increased, although these observations were not statistically significant (Fig. 2A-C; Fig. S3). Lastly, in FGFR2^E565G^, the phosphorylation of both PLCγ and FRS2α showed a significant increase compared to that in the healthy controls and FGFR2^C342Y^ (Fig. 2B; Fig. S3). Additionally, the PLCγ phosphorylation staining in FGFR2^S252W^ and FGFR2^E565G^ presented with a single band, unlike that in the healthy control samples and FGFR2^C342Y^, which showed additional lower-molecular mass bands (Fig. 2A; Figs S2, S3). The phosphorylation of FRS2α showed an additional band around 120 kDa in healthy control lines and FGFR2^C342Y^, but not in FGFR2^S252W^ and only slightly in FGFR2^E565G^ samples (Fig. 2A; Figs S2, S3).

Additionally, we tested the effects of the patient pathogenic variants on the expression of FGFR2 target genes – *DUSP6*, *ETV4* and *ETV5* – under unstimulated, bFGF-free conditions by RT-qPCR (Fig. S4). All three target genes are signaled through FRS2α and the MAPK pathway (Ornitz and Itoh, 2015). *DUSP6* showed significantly increased expression in FGFR2^C342Y^ cells (∼2-fold) and was downregulated in FGFR^S252W^ and FGFR2^E565G^ cells (∼3- to 10-fold) (Fig. S4A). *ETV4* was only significantly downregulated in FGFR2^S252W^ cells (∼4-fold). *ETV5* was significantly downregulated in FGFR2^S252W^ (∼3- to 5-fold) and FGFR2^E565G^ (∼2.5- to 4-fold) cells (Fig. S4B,C). Together, the expression levels of the FGFR2 target genes show a distinct expression profile in each cell line based on the patient pathogenic variant they harbor.

## DISCUSSION

In this study, we demonstrate the successful generation of three patient-derived hiPSC lines. These lines each represent a different pathogenic variant in the FGFR2 receptor that is causative of craniosynostosis. The access and availability of these patient-specific *in vitro* disease models are important as they recapitulate *FGFR2*-linked craniosynostosis, with the cellular context and genetic makeup of the patient (Rispoli et al., 2024). The patient-derived hiPSC lines do not make use of transgenic gene expression approaches for the creation of patient-specific pathogenic variants and effectively negate any other confounding effects due to donor variances. By deriving hiPSC lines from patients specifically, a catalogue of craniosynostosis-afflicted autologous pluripotent stem cells lines can be established. The differentiation of these hiPSC lines can be directed towards the affected tissues. This property can be used to screen and develop personalized therapeutics, tailored to the affected protein, in the appropriate genomic and cellular context without the need to obtain specific tissue from the patient (Walia et al., 2012).

Access to a catalogue of patient hiPSCs enables the potential for genome-editing approaches that aim to correct the causative pathogenic variant, which can yield various beneficial applications. Successfully corrected cell lines can provide isogenic control samples to investigate the impact of pathogenic variants within a genetically identical environment, thereby negating possible confounders due to donor variances. Additionally, corrected cell lines can be applied in transplantation purposes that aim to replenish the affected tissues with a healthy population of multipotent or somatic cells (Al Abbar et al., 2020).

The three patient-derived hiPSC lines all demonstrate their pluripotency in stemness by expression of embryonic stem cell markers on both a protein and mRNA level. Moreover, germ layer differentiation was also confirmed by protein and mRNA expression of lineage markers for mesoderm, endoderm and ectoderm. No large CNVs were observed as a consequence of the reprogramming process. The 75 kb duplication and 160 kb deletion observed in FGFR2^S252W^ and FGFR2^E565G^, respectively, were also found in the original patient-derived PBMCs from which the hiPSC lines originate. It is important to consider that genomic aberrations like these are too small to be detected by conventional karyotyping and can therefore be easily overlooked. In our view, the GSA should be included as an hiPSC line quality control to ensure genomic stability of the established patient-derived *in vitro* models.

The chromosome 10 75 kb duplication in FGFR2^S252W^ is located on chr10:135301270-135376669 (GRCh37/hg19), which aligns to the q26.3 arm nearing the end of the chromosome. In contrast, *FGFR2* is located at chr10:123237844-123357972 (GRCh37/hg19), which aligns to q26.13 arm ∼12 million base pairs distance from the duplication. However, currently we do not possess the data to accurately speculate where the duplicated sequence has been inserted in the genome. Within the duplicated region, three genes are located: *SCART1*, *CYP2E1* and *SYCE1*. The *SCART1* and *SYCE1* genes have no published research associated with craniosynostosis, bone homeostasis or any of the pathways investigated in the study. Although *CYP2E1* is mentioned in several studies of bone homeostasis, these studies investigate the effects of alcohol on bone loss and repair (Chakkalakal et al., 2005; Chen et al., 2006). Furthermore, a single mouse study has found upregulation of *Cyp2e1* in calvaria bone tissues of osteogenesis imperfecta mice (Moffatt et al., 2021), but no data on *Cyp2e1* and craniosynostosis are present. The 160 kb deletion in FGFR2^E565G^ is located on chr13:42412102-42571599 (GRCh37/hg19). Within this area, a single gene – *VWA8* – is located. However, no published research on *VWA8* associates this gene with bone homeostasis or the pathways investigated in this study. Overall, neither the 75 kb duplication, nor the 160 kb deletion, is likely to impact the results on FGFR2 observed in the data and in association with craniosynostosis. However, we cannot rule out an interaction of these genes with FGFR2 in craniosynostosis on the basis of our current observations.

With the successful generation of the hiPSC lines, we aim to explore the impact of the patient pathogenic variants on the FGFR2 protein and its direct docking proteins. We wanted to observe the intrinsic consequences of the pathogenic variants on the protein signal transduction without any interference from ligand stimulation. Therefore, we opted to compare the three mutant FGFR2 proteins with the WT FGFR2 protein in its inactivated state in which no ligand is bound to the receptor. First, we compared our own findings to the data and claims in published articles (Ahmed et al., 2008; Anderson et al., 1998; Chen et al., 2007; Eswarakumar et al., 2006; Liu et al., 2013; Mangasarian et al., 1997; Mansukhani et al., 2000; Miraoui et al., 2009; Mohammadi et al., 1996; Neilson et al., 1995; Suzuki et al., 2012; Yu et al., 2000) that make use of a non-human *in vitro* model or transgenic expression of mutant FGFR2. Second, we report novel findings on the effects of each of the patient pathogenic variants on FGFR2 receptor phosphorylation and the associated docking proteins PLCγ and FRS2α in the absence of stimulating ligand. From published literature (Mohammadi et al., 1991; Peters et al., 1992), it is known that the docking protein PLCγ is able to bind to the intracellular c-terminal tail of FGFR2 with high affinity once the receptor is activated by phosphorylation. In turn, PLCγ is activated and mediates downstream signaling through the PKC pathway (Ornitz and Itoh, 2015). The docking protein FRS2 has been reported to colocalize with FGFR2 prior to stimulation in healthy cells, but requires phosphorylation of specific residues to further activate downstream signaling pathway such as PI3K and MAPK (Ahmed et al., 2010; Ornitz and Itoh, 2015) (Fig. 2D).

The results in this study show three distinct signaling profiles in patient hiPSC lines as a consequence of different FGFR2 gain-of-function variants. In an unliganded state, WT FGFR2 illustrates a minimal level of receptor phosphorylation, which is amplified once ligand binds to the receptor (Ahmed et al., 2010). FGFR2^C342Y^ already shows increased FGFR2 phosphorylation despite the bFGF-free culture conditions, confirming the reported ligand-independent gain-of-function of the C342Y pathogenic variant (Neilson and Friesel, 1995). However, it must be stated that the quantification of the western blot data presented in this study is able to only show a significant increase in FGFR phosphorylation compared to WT control 1 and not to WT control 2. The difference between the WT controls may be explained be donor variability. However, the underlying characteristics responsible for these differences between controls remain unclear, and any potential explanation is purely speculative. Nevertheless, we believe that the absence of significance between WT control 2 and the C342Y-carrying line does not impact the interpretation and conclusions of our study. Moreover, this discrepancy supports the notion of the importance of isogenic controls.

We tested expression of three FGFR2 target genes – *DUSP6*, *ETV4* and *ETV5*, which are canonically activated by MAPK-ETS signaling (Ornitz and Itoh, 2015). We speculated that the *FGFR2*-C342Y pathogenic variant would result in regulation of these target genes as this variant leads to ligand-independent autophosphorylation of the receptor. This regulation is observed for *DUSP6*, but not for *ETV4* and *ETV5*, which were unchanged. The *DUSP6* observation is in line with the autophosphorylated state of the mutated FGFR2. However, the docking proteins PLCγ and FRS2α are not phosphorylated in this condition, and this is in line with observations of unchanged expression of *ETV4* and *ETV5* compared to that in control cells. This shows that the molecular phenotype of this craniosynostosis variant is more complex, thereby underscoring the importance of variant-specific hiPSC craniosynostosis disease models. This notion is further supported by the expression analyses in the FGFR2^S252W^ and FGFR2^E565G^ hiPSC lines, which present their own complex expression profile in the absence of ligand. Thus, these findings support the notion that our hiPSC models are powerful tools to unravel molecular and phenotypic heterogeneity of craniosynostosis.

FGFR2^C342Y^ shows an additional lower-molecular mass fragment, which seems strikingly less present in the other healthy control and patient samples. This additional fragment is most likely corresponding with a less glycosylated form of the FGFR2 protein resulting from disruption of one of the glycosylation sites by the C342Y pathogenic variant (Ahmed et al., 2008; Hatch et al., 2006; Mangasarian et al., 1997; Polanska et al., 2009). This hypothesis can be validated by experimentally reproducing the ^35^Si pulse-chase experiment described in Mangasarian et al. (1997) or the endoglycosidase H digestion described in the study Hatch et al. (2006) with heathy control and FGFR2^C342Y^ hiPSC lines.

In our study, we found that neither PLCγ nor FRS2α phosphorylation is significantly altered in the FGFR2^C342Y^ samples. Interestingly, this suggests that despite FGFR2 being constitutively phosphorylated, the *FGFR2*-C342Y pathogenic variants does not activate downstream signaling through PLCγ or FRS2α in unstimulated conditions. In non-mutated conditions, FGFR2 is activated by ligand binding, initiating an autophosphorylation cascade and subsequently activating the docking proteins (Ornitz and Itoh, 2015). However, through a yet unknown mechanism, C342Y-mutated FGFR2 is able to become phosphorylated but unable to activate its docking proteins PLCγ and FRS2α. We suspect that this mechanism involves the conformational changes that occur once ligand binds to FGFR2 (Farrell and Breeze, 2018; Lin et al., 2012). A strong player in the conformational change process and mediation of downstream signaling appears to be growth factor receptor-bound protein 2 (GRB2) (Ahmed et al., 2010; Timsah et al., 2014). Therefore, we postulate that the effects we observe by the pathogenic variants on the receptor can be influenced by the conformation of the receptor protein in combination with the state of GRB2.

In contrast to FGFR2^C342Y^, both FGFR2^S252W^ and FGFR2^E565G^ seem to maintain their minimal FGFR2 phosphorylation state under bFGF-free conditions, similarly to the healthy control lines. For FGFR2^S252W^, this would be in line with published research as it has been observed that the pathogenic variant increases affinity and decreases specificity for FGF ligand (Anderson et al., 1998; Yu et al., 2000).

Ahmed et al. (2008) showed that in HEK293T cells expressing WT FGFR2, FRS2 is minimally phosphorylated. However, cells expressing S252W-mutated FGFR2 present increased FRS2 phosphorylation in unstimulated conditions (Ahmed et al., 2008). These observations are in line with our results that show, although non-significant, a trend for increased FRS2α phosphorylation in the FGFR2^S252W^ hiPSC line under bFGF-free conditions. In addition, that same study shows that S252W-mutant FGFR2-expressing cells display increased clustering of FGFR2 on the cell membrane, which is not seen when WT FGFR2 is expressed (Ahmed et al., 2008). This suggests an active interaction between FRS2 and unstimulated S252W-mutated FGFR2 protein through a mechanism that is not yet fully elucidated but could also be at play in the FGFR2^S252W^ patient line. Furthermore, PLCγ phosphorylation appears to be substantially increased in FGFR2^S252W^ samples. The *FGFR2*-S252W pathogenic variant has been previously reported to promote PKCα activity in murine C3H10T1/2 cells, downstream of PLCγ. This observation pointed towards a regulatory role of PLCγ in osteogenic differentiation induced by the *FGFR2*-S252W pathogenic variant (Miraoui et al., 2009). Together with the data presented in this study, we suspect that *FGFR2*-S252W-mutated hiPSCs may already be primed to promote FGFR2 signaling through the PLCγ pathway without the presence of activating ligand. However, follow-up research investigating the behavior of mutant FGFR2 signaling when ligand is present has yet to be performed with the cells described in this study.

Intriguingly, the data on FGFR2^E565G^ phosphorylation presented in this study do not fully corroborate previously published findings. Unlike the other patient pathogenic variants described in this study, the E565G pathogenic variant is located on the intracellular tyrosine kinase domain of FGFR2 (Kan et al., 2002). The E565G pathogenic variant is described to disengage a molecular brake in the hinge region of the tyrosine kinase domain of FGFR2 and enables ligand-independent autophosphorylation of the receptor and heightened peptide substrate phosphorylation, similarly to the C342Y pathogenic variant (Chen et al., 2007). However, we observed no significant increase in FGFR phosphorylation while under bFGF-free conditions in the FGFR2^E565G^ lines. The discrepancies in data may originate from the lack of cell models in which the molecular mechanism of the E565G pathogenic variant was investigated in previously published articles. The published research makes use of E565G mutant and WT FGFR2 expressed and purified from a baculovirus/insect cell system. Purified FGFR2 proteins were analyzed with various *in vitro* proteomic techniques to ascertain mutant FGFR2 properties and interactions (Chen et al., 2007; Mohammadi et al., 1996). In contrast, our findings are based on an *in vitro* cell culture model that retains the molecular makeup of the cells and includes potentially critical interactions of FGFR2 with other molecular or cellular players.

However, it is largely unknown how E565G-mutated FGFR2 interacts with PLCγ. Normally, the molecular brake in the tyrosine kinase domain of FGFR2 inhibits phosphorylation and subsequent activation of PLCγ in an unliganded state of FGFR2 (Mohammadi et al., 1996). However, the E565G pathogenic variant is proposed to dislodge this inhibitory brake, activating the receptor ligand independently (Chen et al., 2007). This could then promote the high-affinity binding site for PLCγ without the presence of the ligand. In agreement with this model, our data demonstrate heightened elevation in PLCγ phosphorylation in FGFR2^E565G^ cells not observed in healthy control hiPSCs or the FGFR2^C342Y^ samples. In addition, we observe increased phosphorylation of FRS2α in the FGFR2^E565G^ samples, without any substantial lead in literature to why this might be the case. As such, this gap reiterates the utility our patient hiPSC lines hold, as they can be used to address such literary shortcomings and allow for follow-up investigation in an appropriate model.

In conclusion, this paper describes the generation of three patient-derived *FGFR2*-linked craniosynostosis hiPSCs, each with a different pathogenic mutation in the gene. Each of these gain-of-function variants reveal a distinct effect on the phosphorylation of FGFR2 in craniosynostosis and between individual patients. A schematic overview recapitulating our findings, reinforced with published research, is provided in Fig. 2D. Here, we propose the effect of the patient *FGFR2* pathogenic variants in the unliganded state of FGFR2 and consequently on the interaction and activation of its docking proteins PLCγ and FRS2α in contrast to WT activation of FGFR2 by ligand binding (Fig. 2D). The generation of patient-derived hiPSCs provides a unique opportunity to gain a better understanding of variant-specific *FGFR2*-linked craniosynostosis at a molecular level in the patient-specific genomics context and illustrates the necessity for the development of personalized therapies.

## MATERIALS AND METHODS

### Ethical review and patient consent

The Erasmus University Medical Center Medical Ethical Review Board approved the study (NL60886.078.17) and patient-informed consent was obtained. Clinical investigation was conducted according to the principles expressed in the Declaration of Helsinki.

### Generation and culture of hiPSC lines

Patient PBMCs were reprogrammed to hiPSCs using a CytoTune-iPS 2.0 Sendai Reprogramming Kit (Invitrogen #A16517) following the manufacturer's protocol. After reprogramming, hiPSC colonies were picked and expanded individually. Multiple clones were generated from all three patients, of which one clone is reported for each patient. During expansion, hiPSCs were cultured in mTeSR1 (STEMCELL Technologies #85850). After expansion, hiPSCs were cultured on Matrigel (Corning)-coated culture vessels in mTeSR-plus culture medium (STEMCELL Technologies #100-0276). Upon reaching 80% confluency, cells were disassociated using ReLeSR (STEMCELL Technologies #100-0484) and passaged in a 1:6 ratio. Basal phosphorylation states were reached by culturing near-confluency hiPSCs for 24 h in TeSR-E6 (STEMCELL Technologies #05946). After 24 h, hiPSCs were harvested for whole-protein lysates.

Pluripotent control lines consisted of three commercially available HuES lines. These lines can be found under the following Resource Identifications: HuES 2 (RRID:CVCL_B150); HuES 6 (RRID:CVCL_B194); HuES 9 (RRID:CVCL_0057).

Healthy hiPSC control lines 1 (EMC230i) and 2 (EMC238i) are derived from PBMCs of two unaffected donors and consecutively reprogrammed by the Erasmus University Medical Center iPS Core facility. The healthy control hiPSCs were generated and validated following the same protocols as for the three patient hiPSC lines described in this study.

### Germlayer differentiation

Trilineage differentiation of hiPSC cultures was performed using a STEMdiff Trilineage Differentiation Kit (STEMCELL Technologies #05230), following the manufacturer's protocol. After differentiation was completed (day 5 for mesoderm and endoderm lineages, and day 7 for ectoderm lineage), the cultures were fixed with 4% paraformaldehyde (PFA) for immunocytochemistry, and RNA was isolated for RT-qPCR.

### Variant analyses and GSA array

Cells were cultured until confluency on Matrigel-coated culturing plates. Upon confluency, the cells were dissociated with ReleSR and pelleted at 200-300 ***g***. Genomic DNA was isolated from the cell pellets using a Wizard Genomic DNA Purification Kit (Promega #A1120), following the manufacturer's instruction.

Regions of interest were amplified using PCR and purified using a PureLink PCR Purification Kit (Invitrogen #K310001). Purified PCR samples were sent to Eurofins Genomics for Sanger sequencing. By their LightRun Tube standards, 25 ng of purified 300-1000 bp PCR product and 25 pmol of primer (forward) respective to the region of interest (Table S2). Trace files were analysed using A plasmid Editor (ApE).

For the GSA, 200 ng of genomic DNA to a maximum volume of 10 µl was prepared and shipped to the Erasmus Medical Centre core facility ‘The Human Genomics Facility’ following the facility's instructions. Genotyping was performed on the GSA-MD version 3 (Illumina) and array data were processed in GenomeStudio 2.0 (Illumina). A facility-specific cluster file was used to determine genotypes, Log(R) ratio and B-allele frequency values. Standard variant quality control filters (call rate<97.5%, cluster separation<0.27) and sample quality control filters [call rate<99%, Log(R)dev>0.18, gender mismatches] were applied using GenomeStudio. Familial relations and identity checks were performed with KING version 2.3.0. CNV detection was performed using PennCNV (version 8 February 2013), filtering CNV calls based on size (<100,000 bp), number of probes (<10), confidence score (<20) and probe density (>20,000 bp/probe).

### Immunofluorescence

Immunofluorescence staining was performed on 4% PFA-fixed cultures. Briefly, slides were incubated for 10 min with cold methanol and permeabilized with 0.1% Triton X-100 (Merck #1086431000) in PBS for 10 min. The slides were blocked with 1% bovine serum albumin (Roche #10735086001) in 0.05% Tween-20/PBS (Sigma-Aldrich #P1379) for 30 min. Antibodies used for the immunofluorescence and their respective dilution are listed in Table S1. Slides were incubated with primary antibody overnight at 4°C or, alternatively, for 1 h at room temperature. Afterwards, the slides were incubated with secondary antibodies for 1 h at room temperature. Slides were mounted with Vectashield Mounting with DAPI (Vector Laboratories #H-1200) and imaged with a Leica SP5 confocal microscope.

### RNA isolation, cDNA synthesis and RT-qPCR

hiPSCs were cultured until they reach confluency. Three separate cultures were cultured in parallel with each other to generate triplicates (*n*=3). RNA was isolated from cells with a Promera ReliaPrep™ RNA Miniprep system (Promega #Z6010) following the manufacturer's protocol.

Complementary DNA (cDNA) was synthesized using a RevertAid First Strand cDNA Synthesis Kit (Thermo Fisher Scientific #K1621), following the manufacturer's protocol. Briefly, 1 µg RNA was suspended in nuclease-free water and mixed with 50 ng µl^−1^ oligo dTs and 20 ng µl^−1^ hexamer primer, and incubated for 5 min at 70°C. Afterwards, end concentrations of 1 mM dNTP, 1 U µl^−1^ RNAse inhibitor and 10 U µl^−1^ reverse transcriptase enzyme were added to the mix. The solution was incubated for 60 min at 42°C, followed by 10 min at 70°C. Nuclease-free water was added to the cDNA samples to a final volume of 250 µl.

Gene expression of hiPSCs was assessed by RT-qPCR, using GoTaq qPCR Master Mix (Promega #A6002) following the manufacturer's protocol. The primers that were used are listed in Table S2. Gene expression was normalized against glyceraldehyde-3-posphate dehydrogenase (*GAPDH*) expression.

### Western blotting

The experiment was performed using one pre-culture of a singular clone of each of the different hiPSC lines. Then, upon initiating the experiment, three independent wells were seeded in parallel by passaging the pre-culture. The separate wells were cultured and processed for protein isolation individually but identically to each other, in order to generate technical triplicates (*n*=3). The experiment was reproduced once, using a new pre-culture from the same clonal line. Cell cultures at 80-90% confluency were washed once with 1× PBS (Gibco) and disassociated with ReLeSR (STEMCELL Technologies #100-0484). The collected cells were pelleted at 400 ***g*** for 4 min. Cell pellets were dissolved in RIPA buffer (Thermo Fisher Scientific #89900), supplemented with 1× protease inhibitor cocktail (Sigma-Aldrich #I3786-1ML), 1 mM phenylmethylsulfonyl fluoride (Roche #10837091001) and 1 mM natrium orthovandate phosphatase inhibitor (Merck #S6508). Protein yield was measured with a Pierce BCA protein assay kit (Thermo Fisher Scientific #23225). Samples were diluted with 6× Laemmli buffer to a 1× Laemmli buffer solution and boiled at 98°C for 5 min.

For the gel electrophoresis, 15 µg whole-protein lysate was loaded on Mini-PROTEAN TGX Precast gels (Bio-Rad #4561084), stacked at 80 V until bands converged and ran at 120 V in 1× Tris/glycine/SDS running buffer (Bio-Rad #1610732). Proteins were transferred to Amersham Hybond P0.45 PVDF blotting membrane (Cytiva #10600100) using a transfer tank. The transfer was performed at 300 mA for 2 h in 1× Tris/glycine transfer buffer (Bio-Rad #1610734). The membranes were blocked with 5% non-fat milk powder in 1× TBS/0.1% Tween-20. Primary antibodies were diluted as listed in Table S1, and the membranes were stained overnight at 4°C. Secondary antibodies were diluted as listed in Table S1, and membranes were stained for 1 h at room temperature. The membranes were developed using Clarity Western ECL Substrate (Bio-Rad #1705060), and the proteins of interest were detected with a Gel Doc XR System (Bio-Rad). Protein expression was performed with actin as loading control, and the quantifications are corrected to actin as such.

Quantification was done on ECL-developed staining images for the western blot data. Developed images of the western blotting were analyzed in GelAnalyzer 19.1. Lanes were detected automatically to ensure equal surface areas for measurements. Similarly, equal-band areas were manually set for each condition. Background correction was applied by valley-to-valley method. The values of the bands of interest were corrected against the actin loading control values and standardized to EMC230i (WT control 1) values.

### Statistical analysis and compact letter display

Statistical analysis was performed using a one-way ANOVA and Tukey's multiple comparison post-hoc test. Statistical significance is displayed in compact letter display format. Mean values assigned with a common letter are not significantly different by the Tukey multiple comparison post-hoc test at *P*<0.05. Mean values that do not share any letter are significantly different by the Tukey multiple comparison post-hoc test at *P*<0.05 (Piepho, 2018).

## Supplementary Material

10.1242/dmm.052123_sup1 Supplementary information
