# Supplementary material for: Generation of human induced pluripotent stem cell lines from patients with FGFR2-linked syndromic craniosynostosis
Source: Dis Model Mech. 2025 Sep 18;18(10):dmm052123. doi: 10.1242/dmm.052123 (PMC12486208; doi:10.1242/dmm.052123)
Supplement: Supplementary information [file dmm-18-052123-s1.pdf]

A.

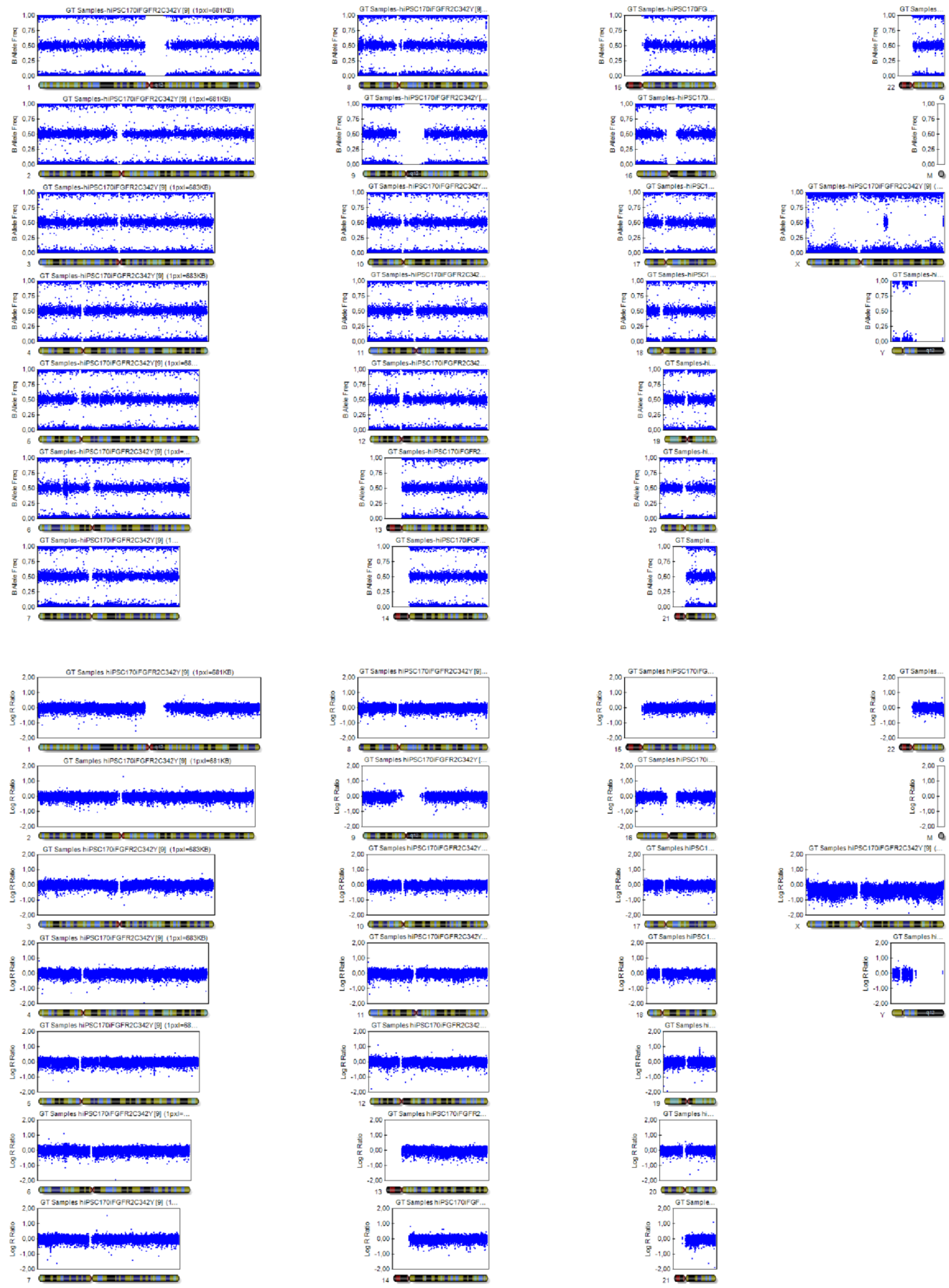

Disease Models & Mechanisms • Supplementary information

B.

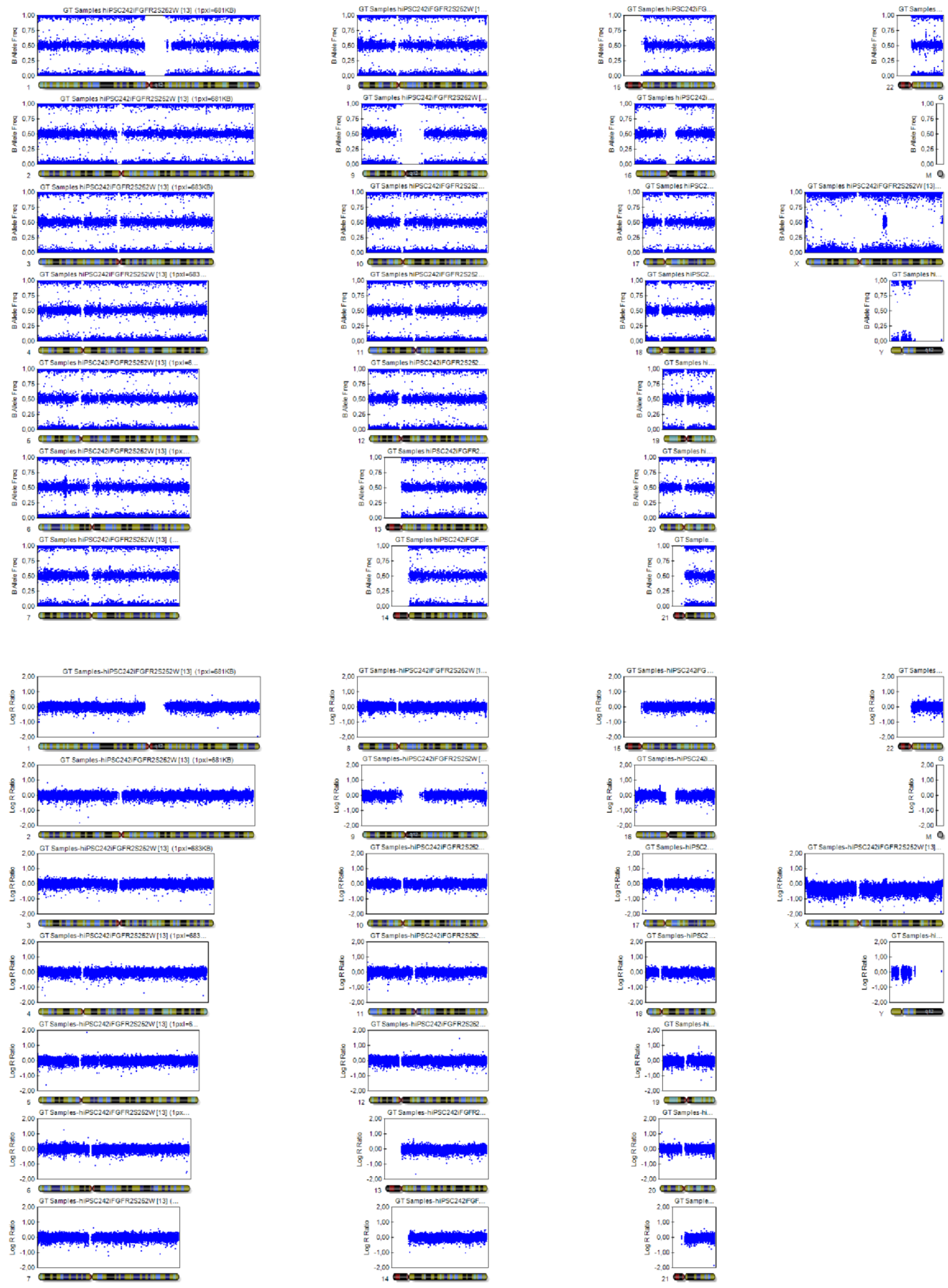

C.

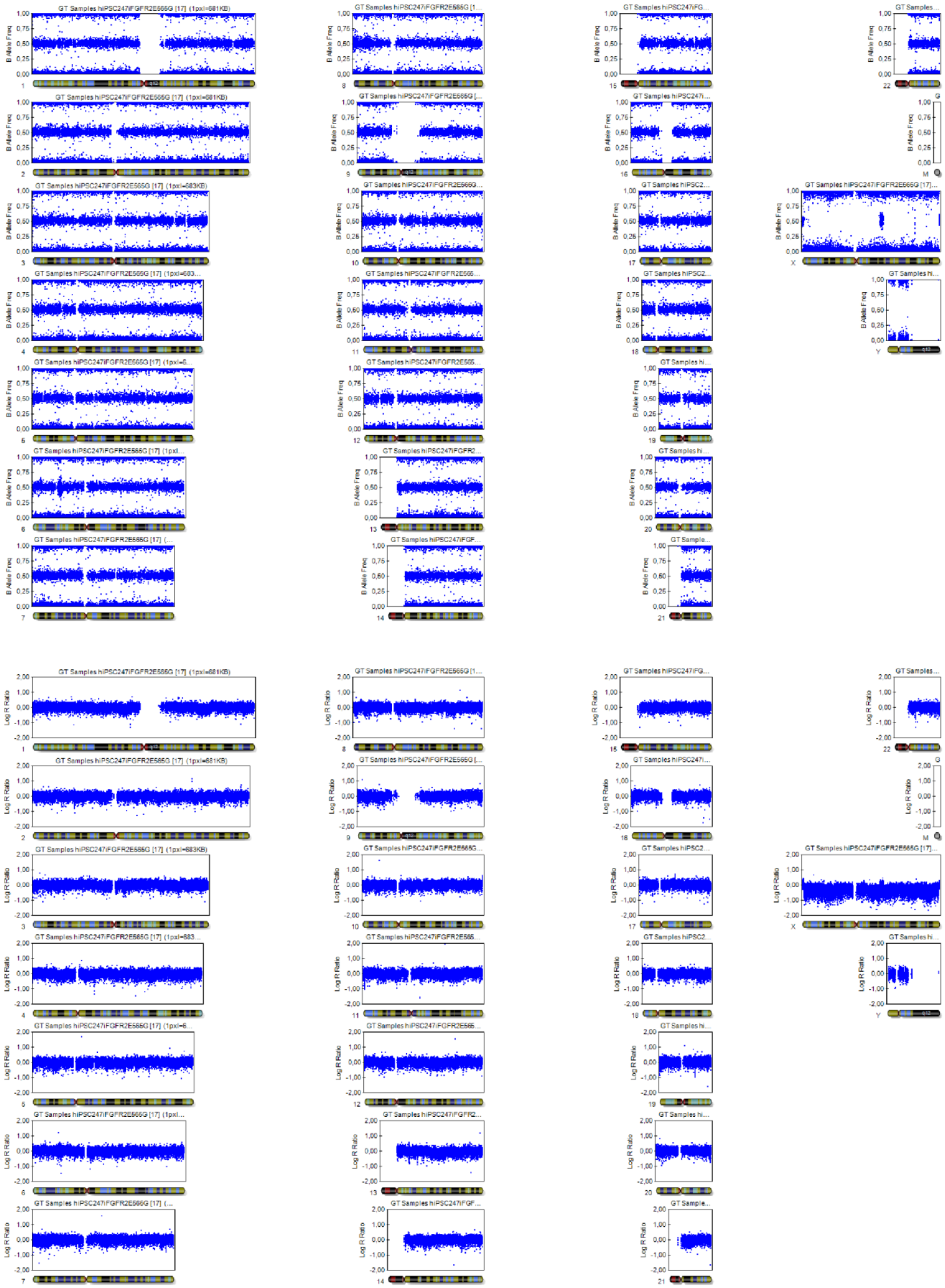

D.

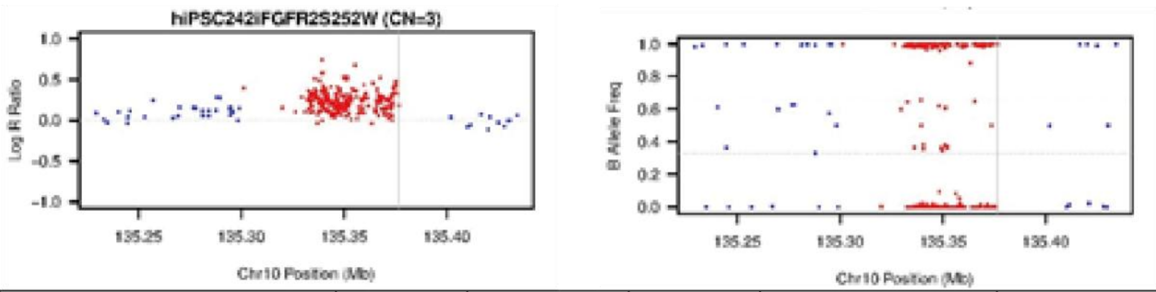

CNV position	#Probes	Length	CN	Sample ID	Confidence
chr10:135301270-135376669	241	75,400	3	hiPSC242iFGFR2S252W	226.646

E.

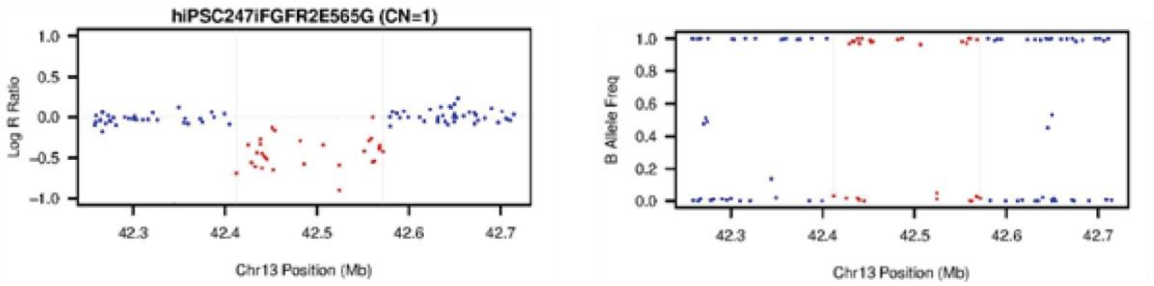

CNV position	#Probes	Length	CN	Sample ID	Confidence
chr13:42412102-42571599	28	159,498	1	hiPSC247iFGFR2E565G	93.68

F.

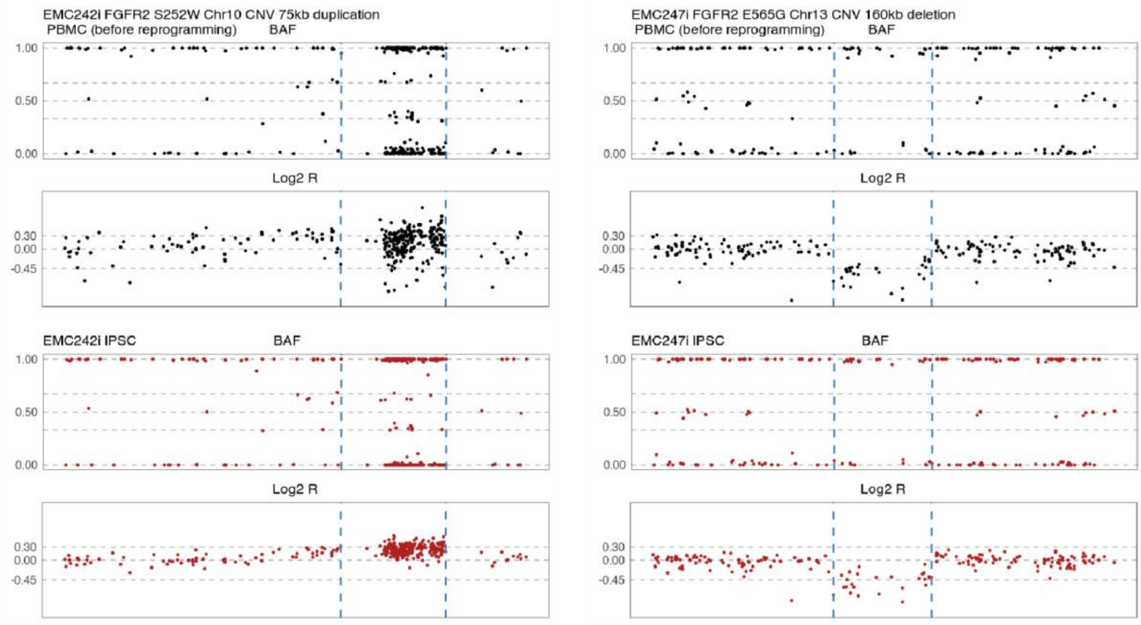

Fig. S1. Additional details on the Global Screening Array analysis. Genomic stability of the patient-derived hiPSCs **(A)** FGFR2^{C342Y}, **(B)** FGFR2^{S252W}, and **(C)** FGFR2^{E565G} through Global Screening Array (GSA) by B-Allele frequency (BAF; upper panel) and LogR Ratio (LRR; lower panel). The BAF is a normalized metric that indicates the presence and proportion of a respective SNP on an allele, where 0 represents the absence of the SNP on both alleles, 0.5 represents the presence of the SNP on one of the alleles (heterozygous), and 1.0 represents the presence of the SNP on both alleles. The LRR is a log-transformed ratio of the measured SNP signals where 0 represents the expected intensity of a wildtype condition of two allele copies. Lower values indicate deletions and higher values indicate duplications. Each window in a panel (either BAF or LRR) represent chromosomes 1 to 22, Mitochondrial DNA (M), X-chromosome (X), and Y-chromosome (Y). **(D)** A close-up view on the observed 75KB duplication (red) on chromosome 10 in FGFR2^{S252W} and a table of details on the variation. **(E)** A close-up view on the observed 160KB deletion (red) on chromosome 13 in FGFR2^{E565G} and a table of details on the variation. **(F)** A side-by-side comparison of the GSA results on the Peripheral Blood Mononuclear cells (black) of which the hiPSCs are derived and the patient-hiPSC lines (red) for FGFR2^{S252W} (left) and FGFR2^{E565G} (right). The hiPSC GSA results (red) are plotted from the same data set as when plotted in D and E.

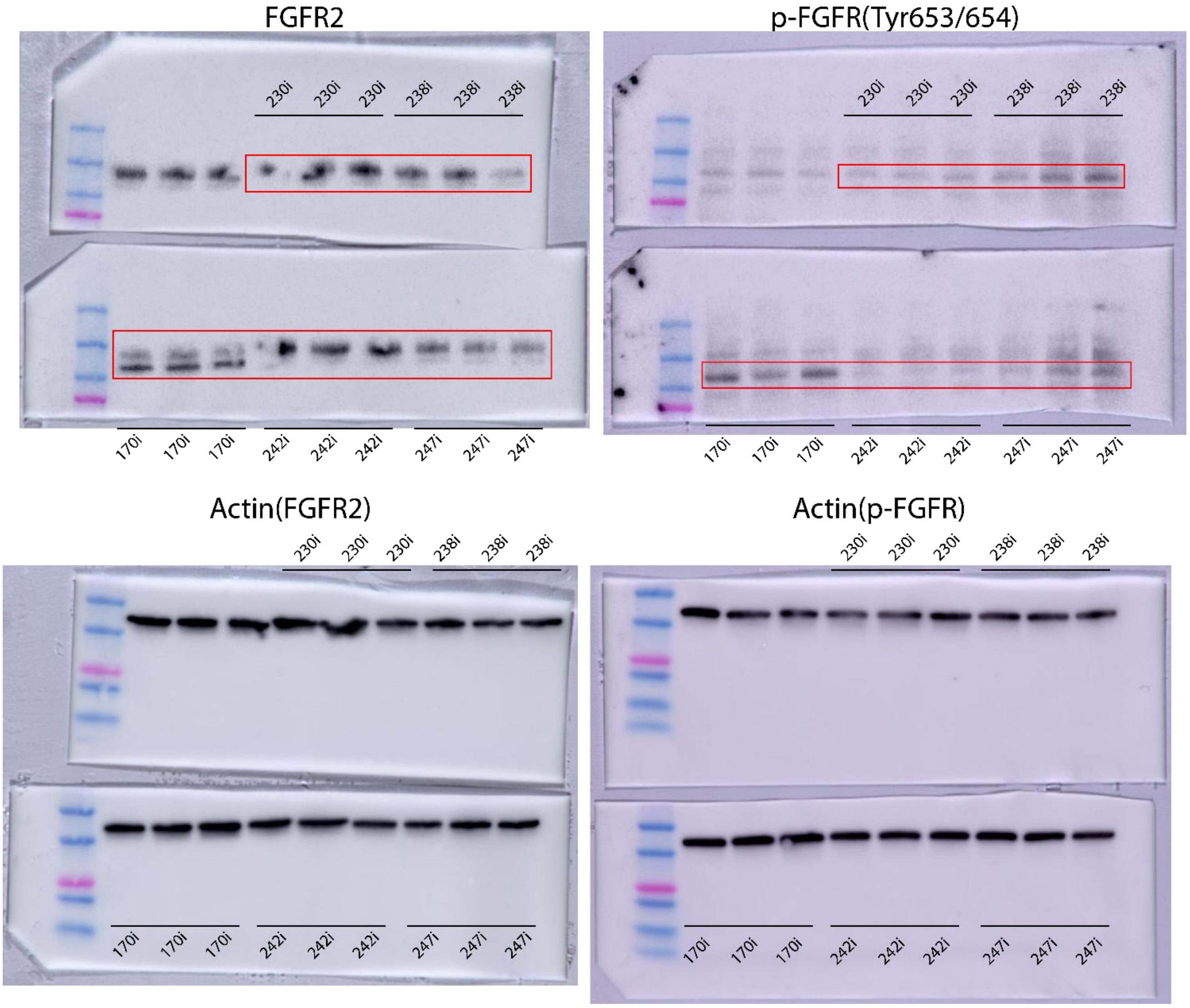

Disease Models & Mechanisms • Supplementary information

PLCy

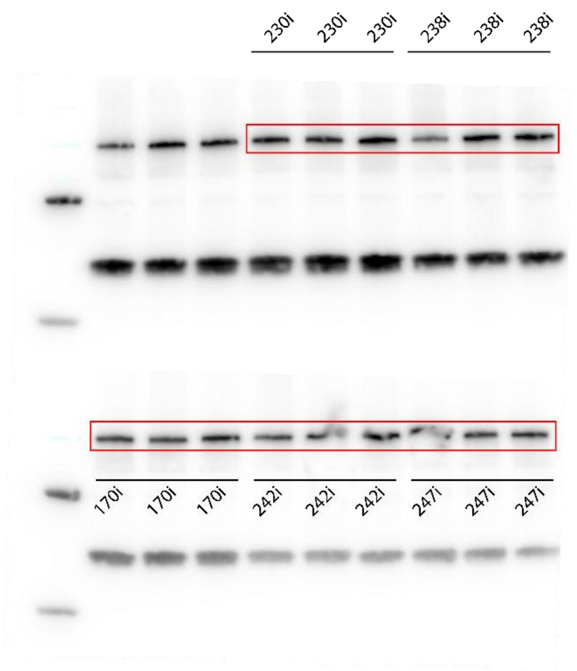

p-PLCy(Tyr783)

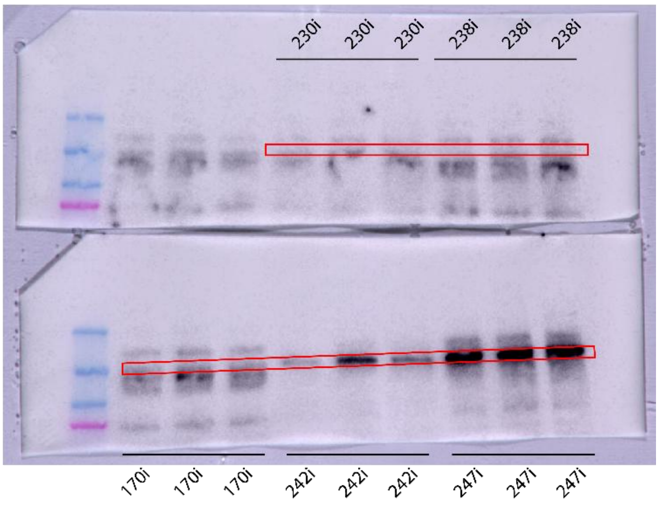

Actin (p-PLCy)

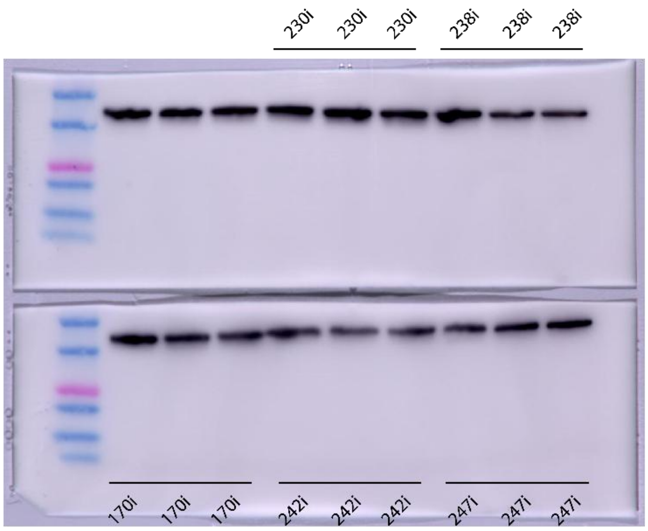

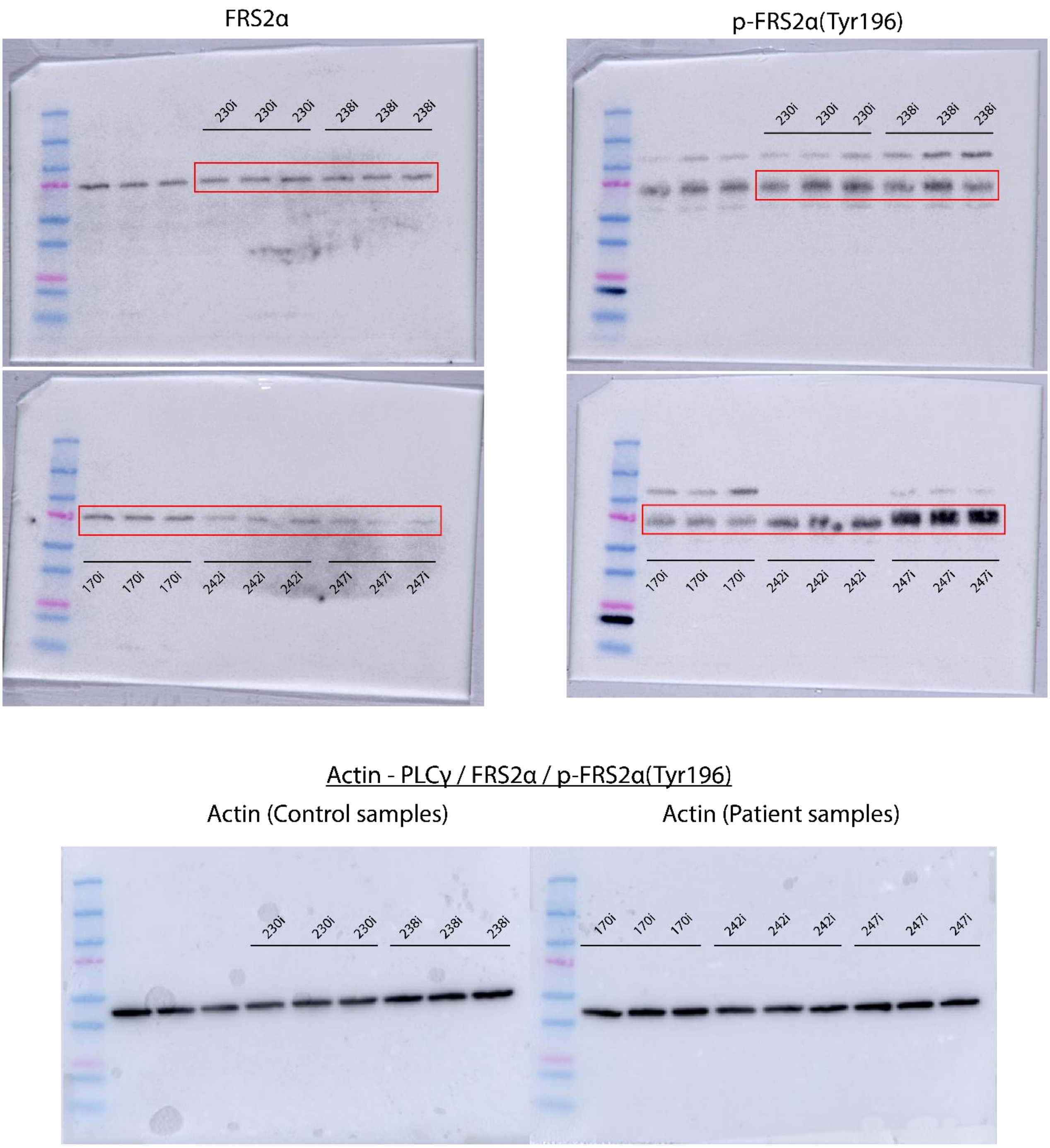

Fig. S2. Unprocessed Western Blot membrane chemiluminescence images. The area used for the quantification is indicated by red rectangles. Healthy control samples 1 and 2 are termed 230i and 238i, respectively. FGFR2^{C342Y} Crouzon samples are termed 170i. FGFR2^{S252W} Apert samples are termed 242i. FGFR2^{E565G} Crouzon/Pfeifer samples are termed 247i.

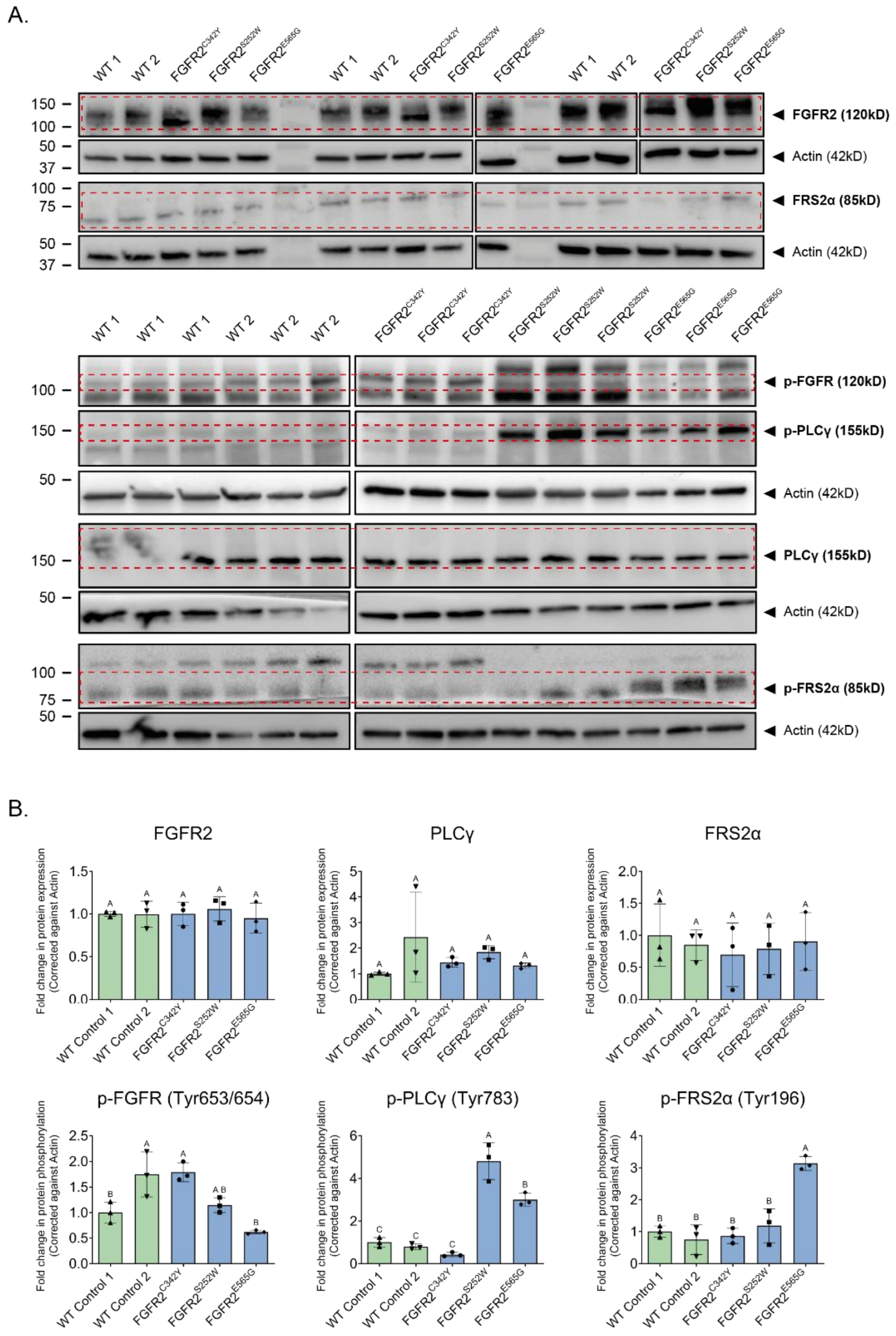

Fig. S3. Reproduction experiment of the data in Fig. 2. (A) Processed Western Blot analysis of whole protein expression and phosphorylation of FGFR2 and docking proteins PLCγ and FRS2α in two healthy control and three patient hiPSC lines. Protein targets are stained with their respective first and secondary antibody and detected by chemiluminescence imaging. **(B)** Quantification of the respective protein or phosphorylation targets in fold change relative to WT control 1. The band intensity was determined for each target, corrected to their respective Actin staining, and normalized to the band intensity of WT control 1. Statistical analysis was performed using One-way ANOVA and Tukey's multiple comparison post-hoc test. Technical replicates used in each cell line tested n = 3. Error bars display mean values with Standard Deviation applied. Statistical significance is displayed in Compact Letter Display format, with the significance threshold of $p < 0.05$.

Disease Models & Mechanisms • Supplementary information

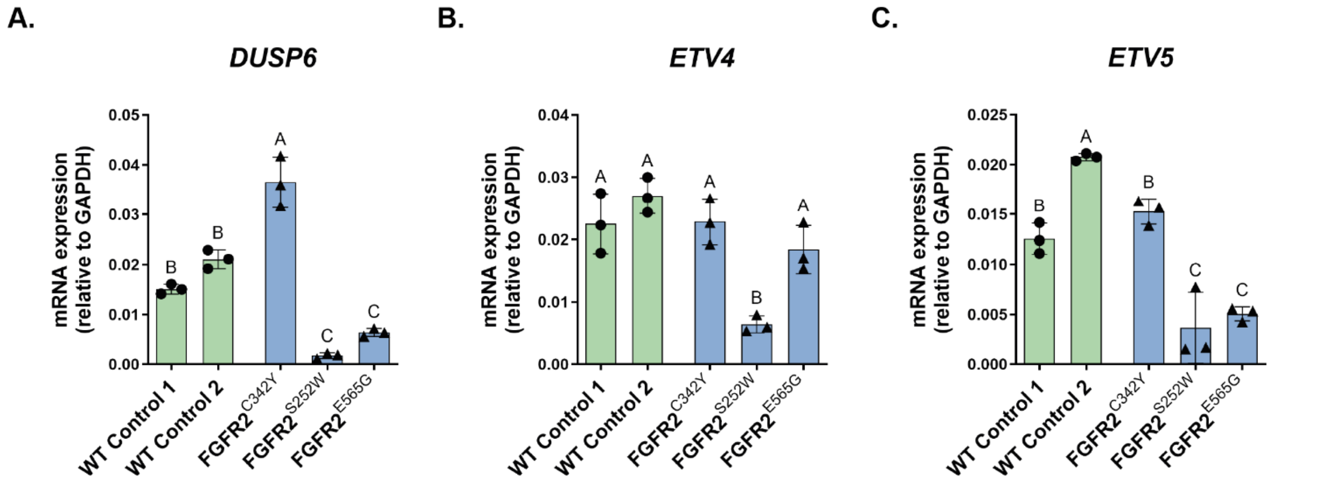

Fig. S4. Gene expression of three FGFR2-target genes assessed by RT-qPCR. mRNA expression of *DUSP6* (**A**), *ETV4* (**B**), and *ETV5* (**C**), are shown in healthy WT control samples and the three patient hiPSC lines. Relative mRNA expression was compared to the GAPDH as a reference gene. Statistical analysis was performed using One-way ANOVA and Tukey's multiple comparison post-hoc test. Technical replicates used in each cell line tested n = 3. Error bars display mean values with Standard Deviation applied. Statistical significance is displayed in Compact Letter Display format, with the significance threshold of $p < 0.05$.

Disease Models & Mechanisms • Supplementary information

Table S1. List of antibodies used in the experiments, dilutions ratios, and their respective suppliers. IF: Immunofluoresence. ES: Embryonic Stemcell.

Applied as	*Antibody*	*Dilution*	*Supplier*
**IF primary antibodies**			
ES Marker staining	Mouse-α-SSEA4 IgG	1:75	Abcam #AB16287
	Rabbit-α-NANOG IgG	1:75	Abcam #AB21624
	Mouse-α-TRA-1-81 IgM	1:75	Abcam #AB16289
	Rabbit-α-OCT4 IgG	1:250	Abcam #AB19857
Mesoderm staining	Goat-α-NCAM IgG	1:100	R&D #AF2408
Endoderm staining	Goat-α-SOX17 IgG	1:100	R&D #AF1924
Ectoderm staining	Mouse-α-β-Tubulin IgG	1:1000	Sigma-Aldrich #T8660
**IF secondary antibodies**			
	Goat-α-Mouse IgG (H+L) Alexa-546	1:500	Invitrogen #A-11003
	Goat-α-Rabbit IgG (H+L) Alexa-488	1:500	Invitrogen #A-11008
	Goat-α-Mouse IgM (H+L) Alexa-546	1:500	Invitrogen #A-21045
	Donkey-α-Goat IgG (H+L) Alexa-488	1:500	Invitrogen #A-11055
	Goat-α-Mouse IgG (H+L) Dylight-594	1:500	Jackson #115-515-166
**Western Blotting**			
Primary antibodies	Rabbit-α-FGFR2 IgG	1:1000	Cell Signaling #23328
	Rabbit-α-Phospho-FGFR (Tyr653/654)	1:1000	Cell Signaling #3471
	Rabbit-α-β-Actin IgG	1:1000	Cell Signaling #4970
	Rabbit-α-PLCγ1	1:1000	Cell Signaling #2822
	Rabbit-α-Phospho-PLCγ1 (Tyr783)	1:1000	Cell Signaling #2821
	Mouse-α-FRS2 (A-5) IgG	1:500	Santa Cruz Biotechnologies sc-17841
	Rabbit-α-Phospho-FRS2α (Tyr196)	1:1000	Cell Signaling #3864
Seconday antibody	Goat-α-Rabbit IgG HRP-linked	1:1000	Cell Signaling #7074
	Goat-α-Mouse IgG HRP-linked	1:1000	Cell Signaling #7076

Disease Models & Mechanisms • Supplementary information

Table S2. List of primer sequences used for the RT-qPCR and Sanger Sequencing experiments and respective targets.

Used for	***Target***	***Forward/Reverse Sequence 5' - 3'***
RT-qPCR	*Brachyury*	GGATGAAGGCTCCCGTCTC
		GCTGTGATCTCCTCGTTCTGATA
	KDR	CTGGCATGGTCTTCTGTGAAGCA
		AATACCAGTGGATGTGATGGCGG
	FOXA2	TACAGGCGCAGCTACACGCACGCAAAG
		GCGGGGCACCTTCAGGAAACAGTCGT
	SOX17	GCTTTCATGGTGTGGGCTAA
		CGCCTTCCACGACTTGC
	SOX1	GGTCAAACGGCCCATGAACGC
		TCCTTCTTGAGCAGCGTCTTGGTCTT
	PAX6	TTTGCCCGAGAAAGACTAGC
		CATTTGGCCCTTCGATTAGA
	OCT4	GACAGGGGGAGGGGAGGAGCTAGG
		CTTCCCTCCAACCAGTTGCCCCAAAC
	NANOG	CAGCCCTGATTCTTCCACCAGTCCC
		CGGAAGATTCCCAGTCGGGTTCACC
	TERT	CCTGCTCAAGCTGACTCGACACCGTG
		GGAAAAGCTGGCCCTGGGGTGGAGC
	REX1	CAGATCCTAAACAGCTCGCAGAAT
		GCGTACGCAAATTAAAGTCCAGA
	ETV4	AACAGACGGACTTCGCCTAC
		CAGAGAAGCCCTCTGTGTGG
	ETV5	TGGACCACAGCAGCAAACAT
		TGGCAGGGTTCAGACAGTTG
	DUSP6	CATCTCGGATCACTGGAGCC
		AGACACCACAGTTCTTGCCC
	GAPDH	CCGCATCTTCTTTTGCGTCG
		CCCAATACGACCAAATCCGTTG
	SeV	GGATCACTAGGTGATATCGAGC
		ACCAGACAAGAGTTTAAGAGATATGTATC
PCR/Sanger Sequencing	*FGFR2*-Exon7	CTCCGGCAGTCTCCTTTGAA
		GCGCAGCTAAGCCTAAATGTC
	FGFR2-Exon9	CTTCCCTTGTTTTCTAGGCC
		GATCATAAATGTGAGTGTGGG
	FGFR2-Exon12	TGAATTGCCCAAGGGGAGACC
		GGGCTTGATCTAGCAAATGAGC

Disease Models & Mechanisms • Supplementary information
